# 1091. HIV Does Not Increase Risk of Disease Severity in Patients with COVID-19 Infection

**DOI:** 10.1093/ofid/ofac492.931

**Published:** 2022-12-15

**Authors:** Salman Bangash, Adriana Betancourth, Ayesha Khan, Chiu-Bin Hsiao, Cassandra Oehler, Charmaine Clarisse D Abalos

**Affiliations:** Allegheny General Hospital, Pittsburh, Pennsylvania; UTRGV school of medicine at Doctors Hospital at Renaissance, Mcallen, Texas; University of Texas Rio Grande Valley, Edinburg, Texas; Allegheny General Hospital, Positive Health clinic, Center for Inclusion Health, AHN; Drexel University, College of Medicine, Pittsburgh, Pennsylvania; Allegheny General Hospital, Positive Health Clinic, Center for Inclusion Health, AHN, Drexel University College of Medicine, Pittsburgh, Pennsylvania; Allegheny General Hospital, Pittsburh, Pennsylvania

## Abstract

**Background:**

Limited literature on the impact of COVID-19 infection in persons living with HIV infection (PLWHIV) has shown conflicting results, especially on the degree of disease severity and morbidity/mortality risk impact. The advent of vaccinations against COVID-19 infection helped combat this horrific pandemic. However, their efficacy in immunocompromised hosts is understudied. The measurement of anti-spike antibody levels makes them an ideal surrogate to study the efficacy of the immune response conferred after vaccination.

**Methods:**

A quality improvement project was conducted between 03/2020 and 03/2022 at Positive Health Clinic (PHC), a Pittsburgh urban Ryan White-funded HIV clinic, where approximately 930 PLWHIV receive care. PHC patients who tested positive for COVID-19 were followed up for their clinical presentations and outcomes. Goals were to observe longitudinal clinical impact with or without vaccinations; the COVID-19 vaccination acceptance and its serum anti-spike antibody response (AS-Ab); and COVID-19 variants impact on protection by vaccination.

**Results:**

By 02/21, a 70% vaccination rate was achieved for PHC PLWHIV. 136 serum AS-Ab tests were collected at least 28 days after completing the primary vaccination series. Five non-responders were noted (2 renal transplant recipients, 2 with advanced AIDS with uncontrolled HIV, and 1 with liver cirrhosis). Low AS-Ab titers were observed in patients with CD4 < 200 or HIV VL > 200. A total of 109 COVID-19 infections in 105 patients were seen. From 03/20 – 11/21, there were 53 infections noted (45 unvaccinated: 5 with severe disease; 8 vaccinated: 0 severe disease). 58 infections (8 unvaccinated, 2 severe, 50 vaccinated (including four 2nd infections: 0 severe) were observed during 11/221 – 03/22. No mortality was noted.

**Figure 1.**
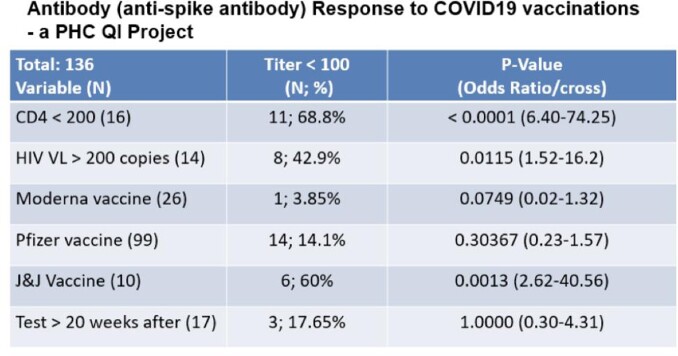

**Figure 2.**
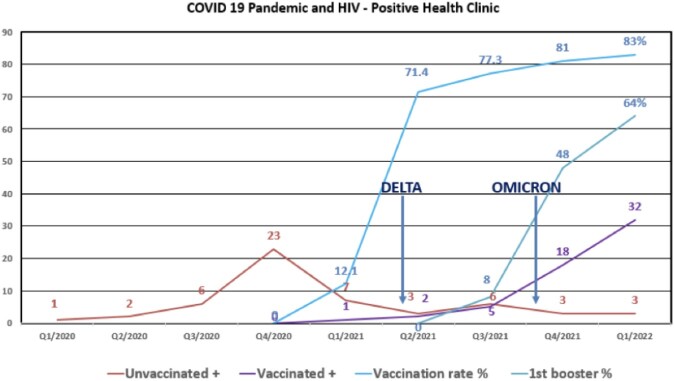

**Figure 3:**
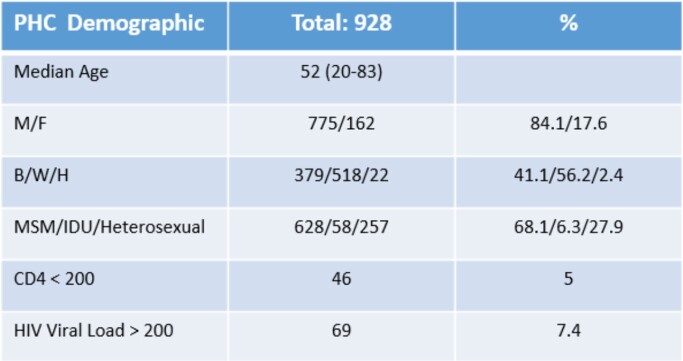
Demographics of PHC

**Conclusion:**

This report did not show mortality directly associated with COVID-19 infection in PLWHIV. Moreover, COVID-19 vaccination is known to reduce the risk and severity of COVID-19 infection in the general population, which was consistent across our study. Interestingly, there were very few COVID-19 infections diagnosed in PLWHIV who had low CD4 count and uncontrolled HIV (CD4 < 200 & HIV VL > 500 copies) even after the surge of the Omicron variant.

**Disclosures:**

**All Authors**: No reported disclosures.

